# Rapid Transcriptional Pulsing Dynamics of High Expressing Retroviral Transgenes in Embryonic Stem Cells

**DOI:** 10.1371/journal.pone.0037130

**Published:** 2012-05-14

**Authors:** Mandy Y. M. Lo, Sylvie Rival-Gervier, Peter Pasceri, James Ellis

**Affiliations:** 1 Developmental and Stem Cell Biology, Hospital for Sick Children, Toronto, Canada; 2 Department of Molecular Genetics, University of Toronto, Toronto, Canada; 3 INRA, UMR 1198 Biologie du Développement et Reproduction, Jouy en Josas, France; International Centre for Genetic Engineering and Biotechnology, Italy

## Abstract

Single cell imaging studies suggest that transcription is not continuous and occurs as discrete pulses of gene activity. To study mechanisms by which retroviral transgenes can transcribe to high levels, we used the MS2 system to visualize transcriptional dynamics of high expressing proviral integration sites in embryonic stem (ES) cells. We established two ES cell lines each bearing a single copy, self-inactivating retroviral vector with a strong ubiquitous human EF1α gene promoter directing expression of mRFP fused to an MS2-stem-loop array. Transfection of MS2-EGFP generated EGFP focal dots bound to the mRFP-MS2 stem loop mRNA. These transcription foci colocalized with the transgene integration site detected by immunoFISH. Live tracking of single cells for 20 minutes detected EGFP focal dots that displayed frequent and rapid fluctuations in transcription over periods as short as 25 seconds. Similarly rapid fluctuations were detected from focal doublet signals that colocalized with replicated proviral integration sites by immunoFISH, consistent with transcriptional pulses from sister chromatids. We concluded that retroviral transgenes experience rapid transcriptional pulses in clonal ES cell lines that exhibit high level expression. These events are directed by a constitutive housekeeping gene promoter and may provide precedence for rapid transcriptional pulsing at endogenous genes in mammalian stem cells.

## Introduction

There is accumulating evidence that gene transcription is discontinuous and occurs as irregular bursts in a pulsatile manner ([1]–[6] reviewed in [7]–[9]). The pulsing kinetics are known to be highly gene-specific [5], and such pulses of transcription can be associated with variations of gene expression in genetically identical populations, potentially giving rise to subpopulations that can respond to developmental cues or environmental stress [7]. Transcriptional pulses can be visualized by the counting of individual mRNA molecules by in-situ hybridization with fluorescent nucleic acid probes, or by real-time imaging of a tagged transcript such as the MS2 system.

The MS2 system is an established tool to track transcription in live cells over time [10]. In this system, the MS2 stem-loop repeat is integrated into a reporter gene or part of an endogenous gene. The fusion protein of EGFP and the MS2 bacteriophage coat protein binds tightly to the MS2 stem-loop RNA, allowing the tagging of nascent transcripts in real time. Sites of transcription, which represent the accumulation of new transcripts, appear as a green fluorescent focal dot in the nucleus. Discontinuous transcription is observed when the fluorescence intensity of the focal dot returns to background level. Using this system, bursts of transcription were detected in several eukaryotic cell types in both reporter constructs [3], [5] and in endogenous genes [2], [6]. Transcriptional pulsing was detected using the MS2 system with the endogenous β-actin (*Actb*) promoter, but not with the CMV promoter in an artificial construct, suggesting that transcriptional pulsing is promoter dependent even amongst gene promoters that are known to express abundantly. Moreover, the duration of these transcriptional pulses or “on" states has been reported to range from 2.5 minutes (*dscA* gene of *Dictyostelium* [2]) to as long as 200 minutes (cyclin D1 promoter in human HEK-293 cells [3]). Of the handful of genes studied using the MS2 method to date, all reported transcriptional pulses are longer than a few minutes, and whether there are shorter transcriptional pulses has yet to be studied. Furthermore, it is still unknown how the dynamics of pulsing at the individual cell level contributes to the overall expression at the population level.

It is well established that retroviral expression in embryonic stem (ES) cells is unstable and silenced over time (reviewed in [11]). Retroviral vectors express only at a fraction of the integration sites in ES cells [12], even though retroviral vectors are known to preferentially integrate into transcriptional units, especially near transcriptional start sites (TSS) with a slight bias for histone modification marks associated with actively transcribing genes [13]–[15]. Proviruses at some integration sites may escape silencing, and other integration sites may display variegation in which single copy EGFP vectors express in some of the progeny cells but are silenced in others [16], resulting in a heterogeneously expressing population. Real-time imaging of retroviral variegation in single ES cells and lineage tracking of their progeny [17] have revealed that fluctuations in EGFP fluorescence not only occur over several generations but also within a single cell. Such protein fluctuations within a single cell over time suggest transgene variegation may be due to changes in the transcriptional states of the transgene, reflected as transcriptional pulsing. Moreover, while transcriptional pulsing had been previously detected at integration sites selected by non-homologous recombination [3], whether pulsing occurs at retroviral integration sites has never been determined. We therefore sought to determine whether such transcriptional pulsing can be detected in retroviral transgenes in ES cells.

In this study, we used the MS2 system to image transcriptional dynamics of transgenes at retroviral integration sites in ES cells. Imaging at high frequency, we discovered rapid transcriptional pulses at two independent highly expressed retroviral integration sites, demonstrating that frequent fluctuations of retroviral transgene transcription occur in ES cells. Such fluctuations were also observed on sister chromatids in cells undergoing replication. Although both clones express at high levels, we observed different transcriptional dynamics at the two integration sites, suggesting that the frequency of transcription and the dynamics of transcription collectively contribute to a similar steady state expression as assessed by flow cytometry.

## Results

### Generation and characterization of cell lines harboring integrated retroviral vector

**Figure 1:**
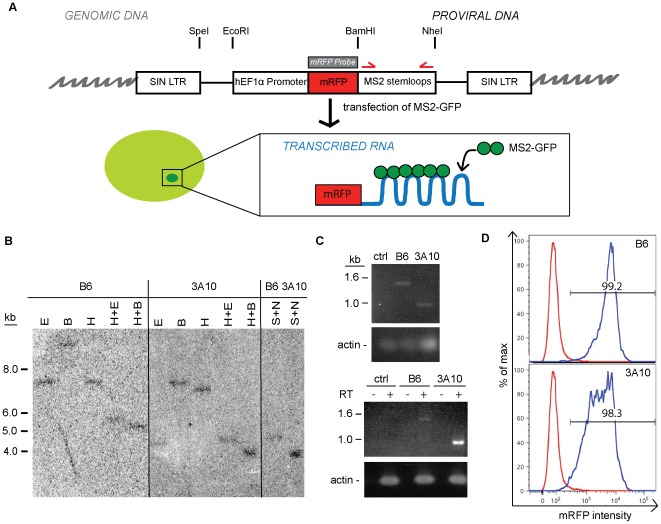
Development of the MS2 system to detect transcription sites of a retroviral transgene. A. Schematic diagram for detecting transcription sites from retroviral transgenes. The MS2 stem-loop was inserted into a HSC1 retrovirus backbone expressing mRFP. The structure of provirus after integration into the genome is shown. Location of restriction enzymes digestion sites, probe used for southern blot analysis (gray box) and primers for PCR (red arrows) to confirm size of stem-loops are indicated. B. Southern blot analysis of genomic DNA from Clone B6 and 3A10 of infected ES cells digested with various enzymes. (E = *Eco*RI, B = *Bam*HI, H = *Hind*III, S = *Spe*I, N = *Nhe*I) Digested DNA was hybridized to the mRFP probe. No *Hind*III site is found within the provirus. C. PCR analysis of number of stem-loops integrated into the genome in the two clones (top) and the number of stem-loops transcribed from each clone (bottom). Uninfected J1 ES cells were used as a control. Actin was used as a loading control. D. Flow cytometry histograms showing expression of mRFP in Clone B6 (top) and Clone 3A10 (bottom). Red line denotes uninfected control J1 cells and blue line indicates clone being interrogated.

In order to determine transcriptional dynamics of retroviral transgenes in ES cells we employed an HSC1-EF1α-mRFP-MS2 vector design. A self-inactivating HSC1 retrovirus backbone with an ubiquitous elongation factor-1α (EF1α) promoter driving the expression of mRFP and the MS2 stem-loop cassette (Figure 1A) was generated. We infected J1 mouse ES cells with this virus at low multiplicity of infection (<0.6) to identify single copy integrants expressing the mRFP-MS2 retrovirus. Two expressor cell lines were isolated through cell sorting for mRFP expression from two independent infections. To confirm both cell lines contained retrovirus inserted as a single copy integrant, southern blot analysis was performed using a probe hybridizing to the mRFP gene. The restriction enzymes *Eco*RI or *Bam*HI cleave the integrated provirus once, and only one band was observed for both clones (Figure 1B). Digestion with *Hind*III, which is not found on the integrated provirus, also yields a single band of different size for both clones, confirming the two proviruses are integrated at different integration sites. Digestion with *Nhe*I and *Spe*I also revealed that the MS2 stem-loop fragment was longer in Clone B6 than Clone 3A10 (Figure 1B). To further confirm this result, PCR was performed on genomic DNA of both clones to examine the number of MS2-stemloops that were successfully transmitted through the retrovirus and integrated into the genome of the clones. A full set of 24 MS2 stem-loops would yield a band of 1.4 kb. The size of the amplicon was 1.4 kb from Clone B6 and 0.9 kb from Clone 3A10, which corresponds to 24 stem-loops and 16 stem-loops respectively (Figure 1C). Since direct repeat sequences are difficult to reverse transcribe intact through retroviral vectors, the reduction of stem-loops in Clone 3A10 is not unexpected [18]. RT-PCR analysis shows that the same number of stem-loops was transcribed into RNA (Figure 1C). Flow cytometry analysis was performed to observe the expression of the provirus in these two lines. Both Clone B6 and Clone 3A10 have >98% mRFP+ cells that express to high levels (MFI = 3523 and 2544 respectively), although Clone 3A10 displayed a wider peak (CV = 94.1) compared to Clone B6 (CV = 60.2), suggesting more cell-to-cell variability in mRFP expression levels (Figure 1D). Such high levels of expression are compatible with detection of transcription foci using the MS2 system.

### Visualization of transcription foci at retroviral integration sites

**Figure 2:**
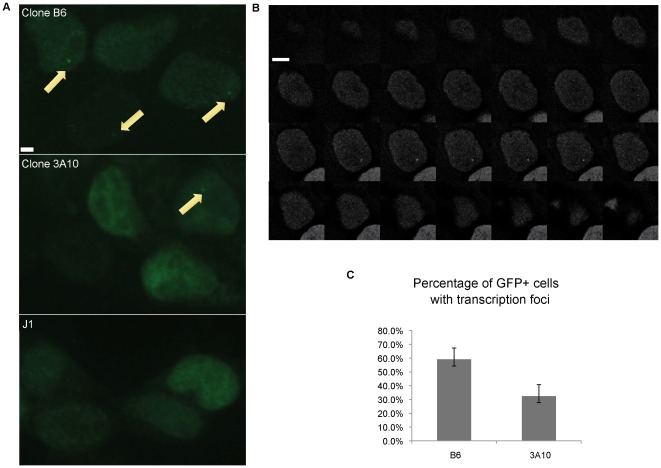
Visualization of transcription foci. A. Transcription foci were detected as focal dots (yellow arrows) in Clone B6 and 3A10 after transient transfection of MS2-EGFP. Uninfected J1 ES cells were used as a control. Scale bar = 2.5 µM. B. Sample images from multiple z-stacks of a cell from Clone 3A10 displaying a focal dot over 6 consecutive focal planes. Z-stacks were 0.3 µM apart. Scale bar = 5 µM. C. Quantification of the percentage of cells with transcription foci in EGFP-positive cells. (n = 2; at least 47 cells were examined for each n).

To determine whether we could detect transcription foci from the mRFP-MS2 retrovirus, we transiently transfected plasmid coding for the MS2-EGFP-NLS fusion protein. A green fluorescent focal dot can be detected in several consecutive focal planes in EGFP-positive cells of Clone B6 and Clone 3A10, but not in uninfected J1 ES cells (Figure 2A), indicating that transcription foci in the system are dependent on the presence of both the mRFP-MS2 virus and the MS2-EGFP-NLS fusion protein (Figure 2A). To further examine the cells, we fixed the two populations 16–18 hours after MS2-EGFP transfection. We collected images in multiple z-stacks to ensure all transcription foci were captured (Figure 2B). We quantified the number of EGFP-positive cells that possessed focal dots and showed that more of these were detected in Clone B6 (59%) than Clone 3A10 (32%) (Figure 2C). Thus, some EGFP-positive transfected cells contained no focal dots, suggesting that these cells were not transcribing the provirus at the time of fixation, which is consistent with the possibility of the provirus being between pulses of transcription. Overall, Clone B6 has a higher frequency of transcription foci correlating with a tighter peak of consistent expression detected by flow cytometry. In contrast, Clone 3A10 has a lower frequency of transcription foci and shows more variable expression by flow cytometry. Since both clones do not have any mRFP-negative cells in the population, these data indicate that the cells without active transcription foci have recently expressed high levels of the provirus in order to maintain such a high MFI of mRFP. We cannot exclude that the cells transcribe at very low levels in this situation, producing undetectable focal dots that are not distinguishable from the nuclear EGFP background.

DNA FISH was performed to ensure that the transcription foci were localized to sites with an integrated provirus. To design a FISH probe, we cloned the integration site using LM-PCR (ligation-mediated PCR). BLAT analysis (http://genome.ucsc.edu) [19], [20] of the LM-PCR product from Clone B6 found the provirus to be integrated in Chromosome 2 (2qH1), in the second intron of *Dlgap4* (Figure S1), a guanylate kinase found to associate with PSD95 at postsynaptic densities of neuronal cells [21]. *Dlgap4* is expressed in ES cells (GEO dataset E-GEOD-21515) [22], and in ES cells this proviral integration site is near regions of H3K4 mono-methylation and H3K20 trimethylation (Figure S1). The provirus in Clone 3A10 is found to be integrated in chromosome 7 in the first intron of hypothetical gene C030039L03Rik. This integration site is also associated with an enrichment of active histone marks (H3K4 dimethylation and trimethylation) in ES cells (Figure S2). We conclude the integration sites are at active gene loci as expected for a retrovirus.

**Figure 3:**
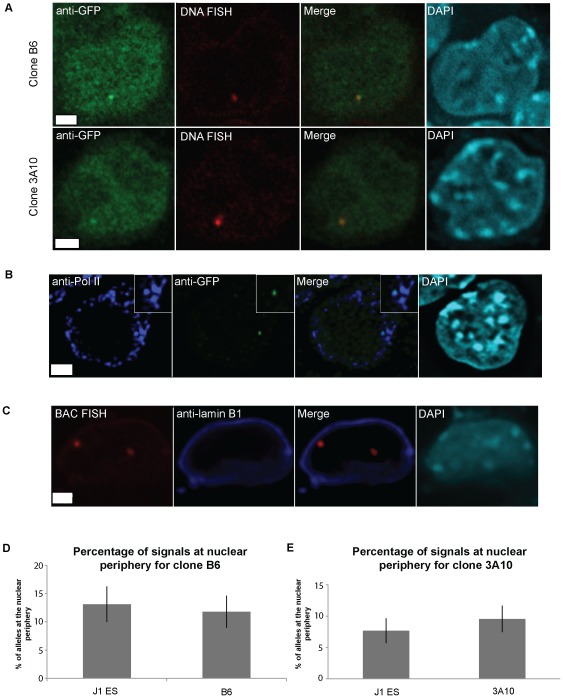
Nuclear positioning of transcriptional foci. A. ImmunoFISH of Clone B6 (top) and Clone 3A10 (bottom) with their respective BAC probes (red) against the integration site, and immunofluorescence staining with EGFP antibodies (green) and DAPI (cyan). A single focal plane with the focal dot is shown. B. ImmunoFISH of Clone B6 cells showing an EGFP focal dot (green) located at RNA Pol II factories (violet) and DAPI (cyan). A single focal plane with the focal dot is shown. Scale bar = 2.5 µM. C. Representative image of *Dlgap4* loci (red) in Clone B6 with respect to the nuclear periphery (violet). The nucleus is counterstained by DAPI (cyan). Scale bar = 2.5 µM. Image shown is the maximal projection of multiple stacks. D. Quantification of the percentage of BAC FISH signals marking the Clone B6 integration site found at the nuclear periphery compared to J1 cells (n = 3, 104–140 FISH signals were counted for each n). E. Quantification of the percentage of BAC FISH signals marking the Clone 3A10 integration site found at the nuclear periphery compared to J1 cells (n = 4, 106–134 FISH signals were counted for each n).

To confirm the integration sites and that the green focal dots observed previously are indeed sites of transgene transcription, a BAC DNA-FISH probe that spans the *Dlgap4* integration site was labeled and immunoFISH performed on Clone B6 using an antibody against EGFP. Transcription foci were marked by EGFP accumulation in a focal dot and were found to colocalize to only one allele of the genomic integration site as expected (Figure 3A). This analysis verifies that the LM-PCR result identified the correct integration site and that the focal dots specifically mark the provirus. A similar result was obtained when DNA FISH was performed on Clone 3A10 using a BAC probe against its integration site (Figure 3A).

Active genes are localized to distinct nuclear subcompartments known as transcription factories [23], which are distinct foci of RNA Pol II. To further confirm that the EGFP focal dot is indeed at sites of transcription, we performed immunoFISH experiments using an antibody recognizing both hyper-phosphorylated and non-phosphorylated RNA Pol II. EGFP focal dots can be found to associate with RNA Pol II staining in 74±3.0% of dots scored in Clone B6 and 74±2.4% of dots scored in Clone 3A10 (n = 3, each n>29 cells), further confirming that focal dots are found at sites of transcription (Figure 3B).

The localization of a gene in the nucleus can be correlated with its gene activity [24]. The nuclear periphery, marked by lamin B1, may act as a transcriptionally permissive or repressive compartment, depending on context [25]–[27]. A transcriptionally active MS2-tagged retroviral vector has previously been observed to be localized near the nuclear periphery at one integration site in lymphocytes [28], [29]. We were also interested in the nuclear positioning of the integration sites in ES cells, and whether the insertion of a transgene alters the positioning of the integration sites. Data from a published database of lamin B1-associated domains (LADs) [30] indicates that neither proviral integration site is associated with the nuclear lamina in ES cells. We confirmed this by performing immunoFISH on uninfected J1 ES cells using BAC probes against the integration sites and anti-lamin B1 antibody (Figure 3C). Alleles are defined as associated to the nuclear periphery if the signal overlaps with lamin B1 staining, which defines the nuclear periphery. We found that less than 13% of the alleles are located at the nuclear periphery for both integration sites (n = 3, 104–140 FISH signals were counted for each n) (Figure 3D,E). This was compared to immunoFISH on the 3A10 and B6 clones in which one of the alleles would contain the provirus, and no difference (P = 0.7 for Clone B6 and P = 0.5 for Clone 3A10, chi-square test) was detected in terms of association to the nuclear periphery (Figure 3D, E). This suggests the insertion of the provirus does not alter the nuclear localization of the integration site.

### Transcriptional pulsing of a retroviral transgene

**Figure 4:**
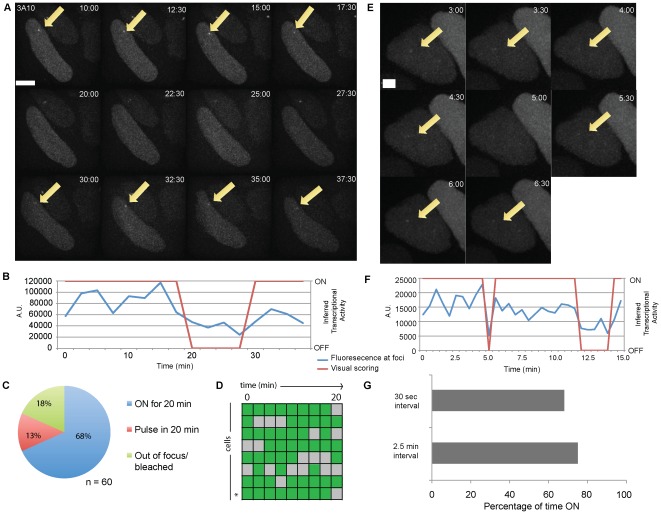
Transcription dynamics of Clone 3A10. A. Representative detection of transcriptional pulses in one cell from Clone 3A10 at 2.5 minute intervals. Transcription foci indicated by arrows. Scale bar = 2.5 µM. B. Transcription foci of the cell depicted in Figure 4A was quantified for EGFP intensity over time. EGFP intensity plots were determined by subtracting background fluorescence at each time point (Blue line). Red line represents visual scoring of inferred transcriptional activity. C. Transcriptional activity of cells possessing EGFP foci at the start of live imaging. D. Summary of transcriptional dynamics displayed by all pulsing cells in Clone 3A10. Green squares indicate timepoints with detectable transcription foci and gray squares represent timepoints without transcription foci. Each square represents 2.5 minutes. The cell shown in Figure 4A is denoted with an asterisk. E. Representative image of transcriptional pulsing of a Clone 3A10 cell recorded at 30 sec intervals. Scale bar = 2.5 µM. F. Intensity time series data (blue line) and visual scoring of inferred transcriptional activity (red line) on the pulsing cell depicted in Figure 4E. G. Cumulative periods of transcriptional activity recorded by the two image intervals. 60 cells were examined for 2.5 minutes interval and 12 cells were examined for 30 seconds interval.

To investigate whether the mRFP-MS2 retrovirus transcribes discontinuously, we performed real time imaging studies to detect transcriptional dynamics. Fields of EGFP-positive Clone 3A10 cells were imaged in 3D-stacks every 2.5 minutes for at least 20 minutes, as transcriptional pulsing could be observed previously with this acquisition setup [2] (Video S1). To increase the efficiency of analysis, we chose fields in which most cells possess transcription foci. While transcription foci were mostly visible throughout the course of imaging, foci were also found to fluctuate in fluorescence intensity, in which optimal levels of transcriptional activity appear as a green focal dot or become undetectable and appear as background, thus reflecting changing levels of transcriptional output (Figure 4A). We scored for the appearance and disappearance of transcription foci that were observed in at least 2 consecutive focal planes. To ensure accuracy in visual scoring, we also quantitated the intensity of fluorescence at the EGFP foci relative to the background fluorescence of a random area in the cell nucleus (Figure 4B). The absence of visible foci coincides with a drop in fluorescence at the focal dot.

To examine if discontinuous transcription is a general phenomenon at the 3A10 retroviral integration site, we imaged 60 cells from this clone and scored for the absence or presence of transcriptional activity over 20 minutes. Cells that contained transcription foci at the start of the imaging period were divided into three groups (Figure 4C) – ones in which the transcription foci are retained constitutively for 20 minutes, ones which display one or more discontinuous transcription pulses within the 20 minutes, and ones in which cells seem to have bleached or the cells have migrated out of the fields or focal planes. As seen from Figure 4C, most 3A10 cells (68%) retained their transcription foci throughout the imaging period. These data suggest the majority of foci do not display pulsing at all, or the provirus is undergoing a very long pulse of transcription in which the start and the end extend beyond the image acquisition period, or that transcriptional pulsing had occurred within the 2.5 minutes interval.

On the other hand, we also detected discontinuous transcription in 13% of 3A10 cells that had an EGFP focal dot at the start of the imaging period. A summary of the recorded transcriptional activity in pulsing Clone 3A10 cells is found in Figure 4D. We defined a “pulse" as an event of gene activity in which the start and the end can be recorded in 20 minutes. As such, only 4 active pulse events were captured in 2 cells and all of the pulses of gene activity were 2.5 minutes. Periods of gene inactivity were also short, ranging from 2.5 minutes to 7.5 minutes with an average of 4.3 minutes. Thus, discontinuous expression is detectable but infrequent in this clone, and only rare active pulses could be documented over 20 minutes.

To examine whether we could detect pulses of transcriptional activity that were shorter than 2.5 minutes, we imaged 3A10 cells and sampled images more frequently at 30 seconds intervals for 15 minutes (Figure 4E,F). While most cells remained transcriptionally active for the duration of the imaging period, discontinuous gene activity was also detected (2/12 cells) (Figure S3). Remarkably, periods of gene inactivity could be seen for as short as 30 seconds (Figure 4E). Similar to cells in this clone that were imaged every 2.5 minutes, pulsing events were observed only in 1/12 cells that were imaged at every 30 seconds. Overall, live cell tracking of 72 cells revealed pulsing events in 3 cells of the 3A10 clone.

We were interested to determine whether imaging at more rapid intervals (ie. 30 seconds) produced similar results as those obtained with the slower 2.5 minutes interval. We compared the cumulative time (Figure 4G) in which the provirus is on or off (as represented by the presence or absence of transcription foci) in the cells which had displayed discontinuous transcription. In the discontinuously expressing cells that were imaged at 2.5 minutes interval, transcription foci were found in 75% of the image capture period, as compared to 68% in the pulsing cells which were imaged at 30 seconds intervals. We conclude that the more rapid imaging frequency can reveal rapid transcription events.

### Detection of Rapid Transcriptional Pulsing

**Figure 5:**
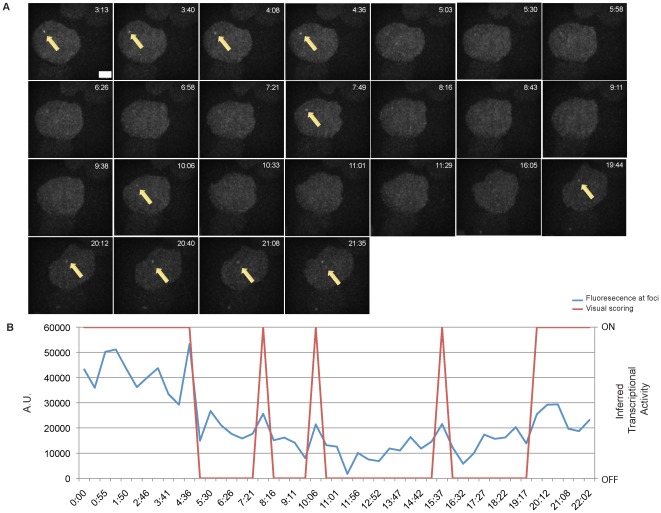
Detection of rapid transcriptional pulsing in Clone B6. A. Rapid and long transcriptional pulses (arrow) detected in a representative cell of Clone B6. Image acquisition was performed every 27 seconds. Scale bar = 2.5 µM. B. Intensity time series data (blue line) of the pulsing cell depicted in Figure 5A. Red line represents inferred transcriptional activity by visual scoring.

To determine whether rapid transcriptional pulsing events can be detected from other integration sites, we performed imaging studies on Clone B6. Because ES cell colonies have different thicknesses but were imaged in constant 300 nm z-dimension slices, each colony has a different number of sections in the z-stack and the thinner colonies can be captured more quickly than thicker ones. Therefore to minimize the image collection time during live imaging experiments, and taking into account the differences in the thickness of the ES cell colonies (12.0 µm to 18.2 µm), the imaging stack was acquired over 25 to 32 seconds (Video S2) for each timepoint. We detected rapid pulses of gene activity or inactivity that persisted only for one frame (ie. 25 to 32 seconds), but also longer pulses that remained for multiple frames as observed from the images (Figure 5A) and from the visual scoring (Figure 5B). We conclude transcriptional pulsing can be detected at two independent retroviral integration sites.

**Figure 6:**
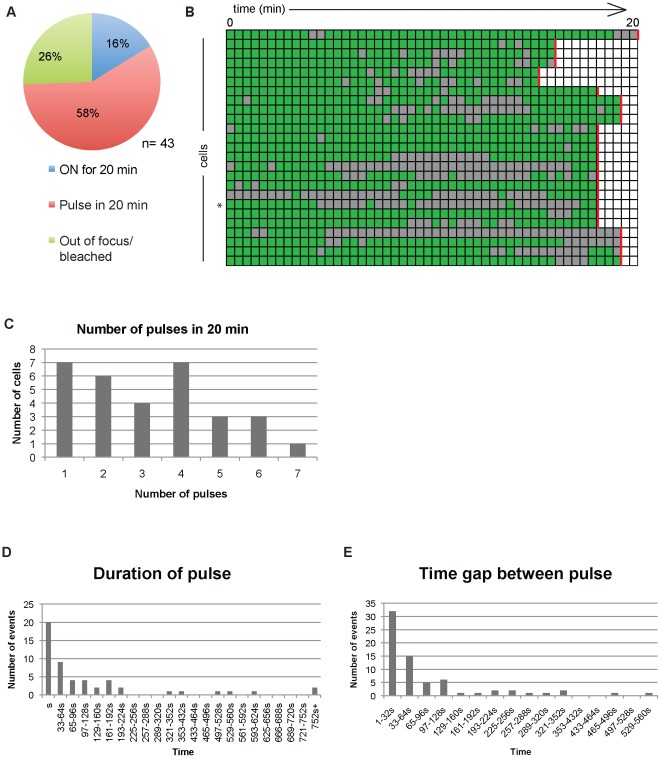
Dynamics of transcriptional pulsing in Clone B6. A. Transcriptional activity of cells possessing an EGFP foci at the start of live imaging. B. Summary of transcriptional dynamics displayed by all pulsing cells in Clone B6. Green squares indicate timepoints with detectable transcription foci and gray squares represent timepoints without transcription foci. Each square represent 25–32 seconds depending on the cell examined and red line denotes 20 minutes of imaging. An asterisk marks the cell shown in 5A. C. Number of pulses detected in 20 minutes in all cells in Clone B6. One pulse represents a cell that is expressed continuously for 20 minutes. D. Distribution of duration of gene activity of Clone B6. E. Distribution of time gaps between transcriptional pulses of Clone B6.

For ease of comparison with Clone 3A10, we limited our analysis of the transcriptional pulses of Clone B6 to the first 20 minutes of image acquisition. Of the 43 Clone B6 cells which possessed a focal dot at the start of the imaging period, the majority (58%) displayed discontinuous transcription, while only 16% remained on for the whole 20 minutes (Figure 6A). A summary of the transcriptional activity of all pulsing B6 cells recorded are shown in Figure 6B. Up to 7 pulses could be detected within the 20 minutes, showing frequent fluctuation between the two transcriptional states (Figures 6C). Most transcriptional events and the gap time between such events were also rapid (<32 seconds), with an average pulse length of 144 seconds (2.4 minutes). The period of time between pulses was also rapid, with an average duration of 88 seconds or approximately 1.5 minutes (Figures 6D,E). The distribution of the lengths of gaps follows that of the Random Telegraph Model [9], in which the gene transitions stochastically between the on and off state. We conclude that rapid pulses of transgene transcription can be detected in both cell clones. The provirus in B6 cells has 24 MS2 stem cell loops and therefore would be expected to have the greatest sensitivity, but most pulses detected were rapid. In contrast, Clone 3A10 has 16 MS2 stem loops but primarily demonstrated longer pulses and gaps. These findings indicate that rapid pulses are not artifacts of reduced sensitivity.

### Transcriptional pulsing of transgenes on sister chromatids

**Figure 7:**
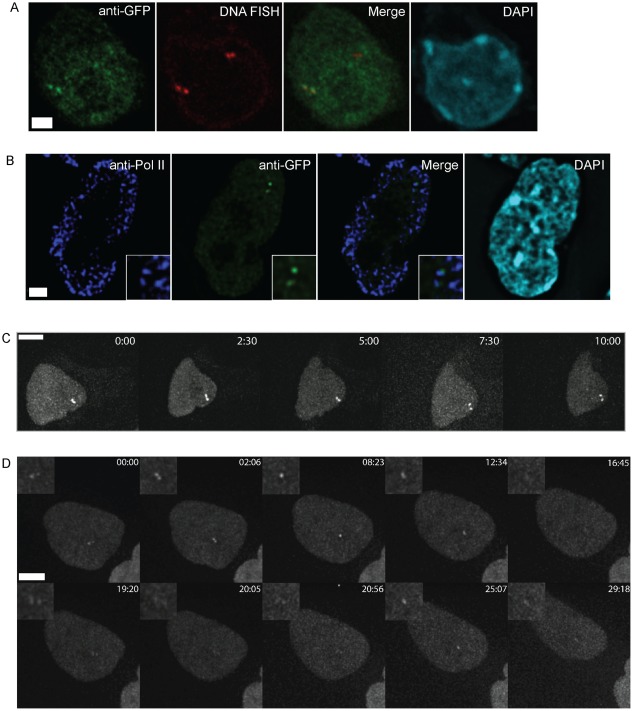
Focal doublets on sister chromatids associate with transcription factories. A. Transcription doublets can be seen in a cell that has undergone replication at the integration site. The pair of transcription foci (green) overlaps the replicated DNA FISH signals (red) on one pair of alleles. The nucleus is counterstained with DAPI (cyan). B. ImmunoFISH of Clone B6 focal doublets (green) show they associate with distinct RNA Pol II factories (violet). A single focal plane with focal doublets is shown. The nucleus is counterstained with DAPI (cyan). Scale bar = 2.5 µM. C. Detection of transcriptional doublets in Clone 3A10 persisting through 10 minutes of image acquisition. Scale bar = 2.5 µM. D. Dynamics of transcription doublets in Clone B6. Scale bar = 2.5 µM.

Pairs of adjacent active transcription sites or “doublets" were previously reported and were interpreted to represent simultaneous transcription sites on sister chromatids [3]. We also observed such focal doublets in our cultures of asynchronously dividing cells. Doublets were found in 32% of Clone B6 cells and 25% of Clone 3A10 cells indicative of transgene transcription from sister alleles in S phase. ImmunoFISH signals of the integration site revealed two pairs of alleles as expected for cells in S phase (Figure 7A). EGFP focal doublets colocalize with one pair of alleles that bear the provirus, confirming that sister chromatids can be co-expressed during S phase. Doublets can also be found to occupy distinct transcription factories, as uncovered by RNA Pol II and EGFP co-staining (Figure 7B). Live imaging revealed that some focal doublets can be observed to persist for at least 30 minutes of imaging (Figure 7C). Remarkably, some doublets also undergo rapid changes, as doublets often rapidly reappear as a single focal dot or disappear synchronously (Video S3). Upon inspection, doublets were also observed in the z-axis, although they may appear as a single focal dot in the xy-plane (Figure S4). The disappearance of the focal doublet is not due to shifting in and out of the sections, as this would be captured by our 3D time-lapse images (Figure 7D, Video S4). These results are consistent with transcriptional pulsing occurring soon after replication, and that both sister chromatids can be transcribed or silenced at the same time. Overall, the dynamics of focal doublets on sister chromatids further corroborates our finding that transcriptional pulses from retroviral transgenes in ES cells can be very rapid.

## Discussion

In this study we used the MS2 system to demonstrate that discontinuous transcription occurs from highly expressed retroviral transgenes in ES cells. We established two ES cell lines transcribing mRFP-MS2 from a strong ubiquitous internal EF1α gene promoter. The most striking feature of both cell clones is that retroviral transgenes can be expressed in very rapid transcriptional pulses in ES cells. The rapid pulses are also observed as focal doublets on sister chromatids that can fluctuate synchronously. Our results reveal that transgenes at different integration sites can employ different transcriptional dynamics to express similar steady-state levels assessed by flow cytometry of mRFP. We propose that high expression levels in any given cell clone ultimately are a consequence of the overall frequency of active transcription foci, combined together with the dynamics of transcriptional pulsing.

### Rapid transcriptional pulsing of retroviral transgenes

The length of transcriptional pulses as well as the time between pulses, has been reported in a variety of cell types and species and range from minutes to hours [2]–[5]. This variation is dependent on 1) the gene examined, 2) the promoter being utilized in the system, and 3) the amount of time in which transcription is followed. When we imaged 3A10 cells at 2.5 minutes intervals, pulses of gene activity and inactivity were as short as 2.5 minutes. This agrees well with the pioneering study of transcriptional pulsing using the MS2 system, in which it was observed that in *Dictyostelium* periods of *dscA* gene activity and inactivity were predominantly short (2.5 minutes) [2]. A more recent study has also shown short fluctuations (within 2.5 minutes) of transcriptional output in a mouse cell line with the MS2 stem-loops knocked into both endogenous β-actin alleles [6].

By imaging both 3A10 and B6 cells at higher frequency, we discovered transcriptional pulses as short as 25 seconds. Our findings extend previous studies that show lentiviral vectors may exhibit discontinuous transcription in Jurkat T cells and 3T3 fibroblasts [5], [31]. These previous studies revealed that transcriptional bursting or pulsing occurs with circadian internal promoters and LTR promoter. Our findings demonstrate that rapid pulses can occur from a highly expressed internal promoter located within an optimally designed self-inactivating retroviral backbone [32]. While higher frequency imaging analysis had previously been performed on transfected reporter genes at 300 ms intervals [3], rapid pulses of gene activity or inactivity were not reported. One factor that may contribute to these differences is that we used the strong ubiquitous EF1α gene promoter integrated into active endogenous genes in ES cells. It is important to acknowledge that we also detected transcriptional events that exceed 20 minutes, providing additional evidence for long pulses in ES cells.

It has recently been shown that Tat-inducible lentivirus in an osteosarcoma cell line did not experience transcriptional pulsing but rather polymerase II consistently transcribed with high elongation rates of up to 50 kb/min at several different single-copy integration sites, in contrast to rates of 1 kb/min when the transgene was present in a multicopy array [33]. It is possible that pulses were not detected because of the excess of Tat transactivator present when these cells are induced, and that the function of Tat is to relieve transcriptional pausing at the HIV-1 TAR sequence [34]. This work emphasizes the importance of studying mammalian cells with single copy transgenes, such as the B6 and 3A10 clones used in our study and in our previous work employing single copy β-globin transgenic mice to investigate Locus Control Region function [35]. In our case, the mRFP-MS2 retrovirus contains a strong internal endogenous EF1α promoter that may still remain subject to pausing, or it may be responsive to other properties that regulate transcriptional pulsing of mammalian promoters in ES cells.

### Transcriptional pulsing of transgenes on sister chromatids

We observed discontinuous transcription in focal doublets expressed from sister chromatids. These results show that pulsing of focal doublets can be rapid but also synchronous and hence are potentially co-ordinated by a shared mechanism. In addition, we observed rapid transitions between focal doublets and a single focal dot within short time frames. Such transitions may represent merging of the focal doublets into a single combined focal dot, or the selective pulsing of only one sister chromatid at a time. The former scenario may suggest rapid movement of the two sister chromatids towards each other, whereas the latter scenario would suggest that pulsing of the two focal dots in a doublet can occur asynchronously.

### Mechanism of rapid transcriptional pulses

We observed rapid transcriptional pulses in 3A10 and B6 cells during interphase and S phase, and periods of gene inactivity were observed over the same time scale. These data suggest that fluctuations in transcriptional output can be very rapid events. Such fluctuations may be due to changes in elongation rates caused by the promoter-proximal pausing of the RNA Pol II complex [36]. A recent study has shown that genes in which RNA Pol II is paused at its 5′ end in ES cells and mouse embryonic fibroblasts (MEFs), are also those that display high RNA Pol II density in the gene body [37]. Due to the size of the RNA Pol II complex, the maximal loading of RNA Pol II is every 75 bp. Stalling of RNA Pol II at the pause site 30–50 bp downstream of the TSS may prevent loading of more RNA Pol II, causing a gap in transcriptional output [38].

Transcriptional pulsing events have been attributed to changes in chromatin state [1], [8]. Turnover of architectural chromatin protein such as histones occurs quickly in ES cells [39] and may play a role in transcriptional pulsing. However, transitions in chromatin state also require co-ordinated changes in histone modifications, and these changes are likely to occur slowly, thus may not play a large role in rapid transcriptional bursts that we observe.

Transcription is known to occur at discrete foci within the nucleus with high concentrations of RNA Pol II, in which multiple genes share transcription factories [23]. Transcriptional pulsing may reflect dynamic association with transcription factories, such that the gene is active only when it is associated with a factory. The rapid pulsing of retroviral transgenes that we observe may reflect the dynamics of their movement in and out of transcription factories. This may readily explain the dynamics of focal doublets during S phase, where the two sister chromatids may share the same factory when they move towards each other. When they are apart, the focal doublets may occupy different factories as we observed. In this case, the simultaneous disappearance of both focal dots in the doublet may represent the sister chromatids moving away from different transcription factories. Alternatively, there may be integration site differences in the types of transcription factories in which the transgene is associated with. While “specialized" transcription factories that express certain subsets of genes have been reported [40], the specific properties of transcription factories have not been described. Different transcription factories may have different transcription properties, which could alter the frequency of transcription foci, or the dynamics of their pulses, depending on integration sites.

### Retroviral Transgene Expression in ES Cells

We were able to detect transcriptional pulses from two high expressing retroviral transgenes. When the pulsing dynamics of the active foci were examined in both cell clones, we found that Clone B6 had few cells with transcriptional pulses of greater than 20 minutes despite the enhanced sensitivity of 24 MS2 stem loops, and that the transgene in most cells pulsed rapidly. At any time point, roughly 60% of B6 cells displayed transcription foci. In order to maintain the consistent levels of gene expression observed by flow cytometry and the absence of mRFP-negative cells in the population, it is likely that all B6 cells must pulse over relatively brief time frames and gaps must be kept short to maximize expression levels.

Clone 3A10 behaved quite differently by having many cells with long transcriptional events of greater than 20 minutes and few with rapid pulses, although the latter may be a consequence of reduced sensitivity conferred by the 16 MS2 stem loops present. We note that Clone 3A10 had a more variable but consistently high expression level, and that only 30% of cells contained transcription foci at any time point. To produce the variable but still high expression, some 3A10 cells must have long pulses to accumulate higher levels of expression, and others may have long gaps or less frequent and shorter pulses which result in reduced levels of expression. This is consistent with the reduced frequency of transcription foci observed in this clone, since cells undergoing a long gap would be observed as not having an EGFP focal dot. However, given that 98.3% of 3A10 cells express mRFP, the 3A10 cells without transcription foci must also activate the transgene with some regularity.

We infer that the overall frequency of active transcription foci, combined with the dynamics of transcriptional pulsing of the active foci, determines the consistency or variability in retroviral expression in a given cell clone. These findings may be informative for deciphering mechanisms of retroviral transgene variegation, which is more challenging to detect using MS2 technology in ES cells because of the low to silent levels of expression produced from these integration sites. Moreover, our use of a constitutive housekeeping human gene promoter inserted into active intragenic integration sites may provide precedence for rapid transcriptional pulsing at endogenous genes in mammalian stem cells.

## Materials and Methods

### Plasmid Construction and Cell Lines

Plasmid HSC1-EF1α-mRFP-MS2 was derived from plasmid HSC1-EF1α-EGFP [32]. The 24 MS2 repeats were isolated from pSL-MS2-24 [10] by *Msc*I and *Cla*I digest. A fragment containing the EF1α gene promoter and mRFP was isolated by *Hind*III digest of HSC1-EF1α-mRFP, followed by blunt ending with Klenow for 30′ and digestion with *Eco*RI. The EF1α-mRFP fragment and MS2 repeats fragment were inserted between the *Cla*I and *Eco*RI sites of HSC1-EF1α-EGFP. Plasmid was further amplified in Stbl2 cells (Invitrogen).

Retroviral production was performed as previously described [41] using the PlatE packaging cell line. Retrovirus was infected into J1 mouse ES cells [42] (gift from En Li). Clonal lines were derived by limited dilution or single-cell FACs sorting for mRFP expressing cells into 96-well plates. Cells were maintained in ES medium media (Dulbecco's Modified Eagle's Medium (DMEM) with 15% fetal bovine serum (FBS) supplemented with 4 mM L-glutamine, 0.1 mM MEM nonessential amino acids, 1 mM sodium pyruvate, 0.55 mM 2-mercaptoethanol (all from Invitrogen) and purified recombinant LIF.

Intactness of retrovirus and clonal independence was confirmed by Southern blotting and PCR using primers ms2-mrfp-fwd2 (AAGCTCCATGGGGACGTCGAC) and ms2-mrfp-rev2 (AACTATAGCTAGCATGCGCAAATTT). Blots were probed with a fragment of mRFP labeled with P^32^ by random priming (Megaprime DNA Labelling System, RPN1606 from GE Healthcare).

### Ligation-mediated PCR

LM-PCR was adapted from a protocol kindly provided by F. Bushman [43]. Genomic DNA was digested with *Mse*I overnight. Digested fragments were ligated overnight by T4 DNA Ligase with 20 µM *Mse*I linker oligos.

Mse1 Linker (+): GTAATACGACTCACTATAGGGCTCCGCTTAA GGGAC;

Mse1 Linker (−): [Phosp]TAGTCCCTTAAGCGGAG[AmC7-Q]

Linker-ligated fragments were digested again with *Pst*I to remove internal fragments. First round of PCR was done using:

linker-primer – (GTAATACGACTCACTATAGGGC) and

HSC1-primer-1 (TCAATAAAAGAGCCCACAACCCCTCAC)

and the following program: 94°C for 1 min, 6 cycles of 94°C for 2 sec and a gradient of 68–71°C for 1 min, 36 cycles of 94°C for 2 sec and 66.2°C for 1 min, 72°C for 4 min and hold at 4°C. 10 uL of each PCR reaction was visualize on an agarose gel to detect a smear of products around 70–100 bp. Products from the first run PCR are diluted and used for nested PCR using:

HSC1-nested-primer2 (GTATTCCCAATAAAGCCTCTTGCT)

and linker-nested-primer (AGGGCTCCGCTTAAGGGAC).

PCR was performed using the program: 94°C for 1 min, 5 cycles of 94°C for 2 sec and 72°C for 1 min, 20 cycles of 94°C for 2 sec and 66.2°C for 1 min, 72°C for 4 min and hold at 4°C. Nested PCR products were transformed into *E.coli* (Invitrogen TOPO TA Cloning Kit) and screened for colonies. DNA was extracted from colonies and digested with *Pst*I to ensure the presence of the cloned inserts. Cloned integration sites were directly sequenced (The Centre for Applied Genomics TCAG) using the M13 forward primer. Successfully cloned integration sites are queried in the mouse genome by BLAT (UCSC Genome Browser) using Feb 2006 NCBI Build 36/mm8 assembly.

### Imaging and image analysis

Imaging was performed on a Quorum spinning disc confocal microscope, composed of a Zeiss Axiovert 200 equipped with a Hamamatsu C9100-13 EM-CCD, Yokogawa scanhead, diode-pumped solid state laser lines (Spectral Applied Research: 405 nm, 491 nm, 561 nm, 638 nm), Ludl motorized XY stage, Improvision Piezo Focus Drive. The equipment is driven by Volocity acquisition software (Perkin Elmer), and powered by a Pentium IV processor.

For live-cell imaging of mRFP-MS2 infected J1 ES cell lines, 5–7×10^5^ cells were transfected with MS2-EGFP DNA and plated on 25 mm round coverslips seeded with E15.5 MEFs and incubated for 16–18 h in ES media. Before imaging, coverslips were washed by PBS and replaced by ES media with no phenol red. Coverslips were placed on an Attofluor cell chamber (Invitrogen, MP 07816) and imaged using 63X water objective (NA = 1.3). Temperature and CO_2_ levels were maintained at 37°C and 5.0% respectively. Images were collected at every 300 nm z-dimension at exposure times of 130 ms to 755 ms depending on the GFP intensities of the field. For image frequency of <32 s intervals, the thickness of captured colonies ranged from 11 µm to 18.2 µm, which is equivalent to 37 to 61 z-focal planes in 25 to 32 seconds. For image frequency of 2.5 minutes intervals, colonies that are up to 28 µm (or 93 focal planes) thick were included. For detection of transcription foci, images were deconvolved using Volocity (Perkin Elmer). Each z-stack of each timepoint was scored for the number of transcription foci, as defined by the most intense signal of at least 4 voxels within the cell. The same imaging setup was used for imaging of fixed cells. Images and videos are extended focus view of the 3D stacks unless otherwise stated.

### 3D DNA Fluorescence in-situ hybridization (DNA-FISH) and immunoFISH

ES cells grown on coverslips with E15.5 MEFs were fixed in 4% PFA/PBS for 10 min. 3D DNA Fluorescence In-situ-hybridization was performed as previously described [26]. BAC clone probes (RP23-450D11 for Clone B6, RP23-423N8 for Clone 3A10) were directly labeled with Spectrum Orange or Spectrum Green by TCAG and hybridized overnight at 37°C. For visualization of EGFP foci post-fixation, Alexa Fluor 488 conjugated rabbit anti-GFP (Invitrogen A21311) or mouse anti-GFP (Invitrogen A11120) was used at 1/1000 dilution. For marking of the nuclear envelope, rabbit polyclonal to anti-lamin B1 (Abcam ab16048) was used at 1/500 dilution. Immunostaining of RNA Pol II foci was performed with mouse anti-RNAP II (clone CTD4H8; Upstate 05-623). Coverslips were counterstained with DAPI and mounted upside down on slides with anti-fade.

## Supporting Information

Figure S1 **Integration site analysis of Clone B6.** Integration site of Clone B6 and neighboring genes (A) with histone modifications present in ES cells and nearby repeat elements (B).
(TIF)

Figure S2 **Integration site analysis of Clone 3A10.** Integration site of Clone 3A10 and neighboring genes (A) with histone modifications present in ES cells and nearby repeat elements (B).
(TIF)

Figure S3 **Transcriptional dynamics of Clone 3A10 imaged at 30 sec intervals.** Summary of transcriptional dynamics displayed by all cells in Clone 3A10 imaged at 30 seconds intervals. Green squares indicate timepoints with detectable transcription foci and gray squares represent timepoints without transcription foci. Cell displayed in Figure 4E is marked by an asterisk.
(TIF)

Figure S4 **Detection of focal doublets in the z-axis.** Focal doublets were detected in the z-axis, while appearing as a single focal dot in the xy-plane.
(TIF)

Video S1 **Time series of Clone 3A10 mRFP-MS2 ES cells transfected with MS2-EGFP at 2.5 minutes intervals.** Images were captured in 3D by a 1.3 NA, 63× objective and total video length is 37.5 minutes. The extended focus image of 64 z-stacks are shown in each frame. Images have been deconvolved with Volocity software. The cell from this video is shown in Figure 4A.
(MOV)

Video S2 **Time series of Clone B6 mRFP-MS2 ES cells transfected with MS2-EGFP at 27 seconds intervals.** Images were captured in 3D by a 1.3 NA, 63× objective and total video length is ∼27.5 minutes. The extended focus image of 61 z-stacks are shown in each frame. Images have been deconvolved with Volocity software. The cell from this video is shown in Figure 5A.
(MOV)

Video S3 **Time series of doublet dynamics in Clone 3A10 ES cells transfected with MS2-EGFP.** Images were captured in 2D by a 1.3 NA, 63× objective, at the speed of 2.16 timepoints per second.
(MOV)

Video S4 **Time series of doublet dynamics in Clone 3A10 ES cells transfected with MS2-EGFP in 3D.** Images were captured in 3D by a 1.3 NA, 63× objective. The extended focus image of 61 z-stacks are shown in each frame. Images have been deconvolved with Volocity software. The cell from this video is shown in Figure 7C.
(MOV)
