# Supplementary figures and images for: Rapid Transcriptional Pulsing Dynamics of High Expressing Retroviral Transgenes in Embryonic Stem Cells
Source: PLoS One. 2012 May 14;7(5):e37130. doi: 10.1371/journal.pone.0037130 (PMC3351450; doi:10.1371/journal.pone.0037130)

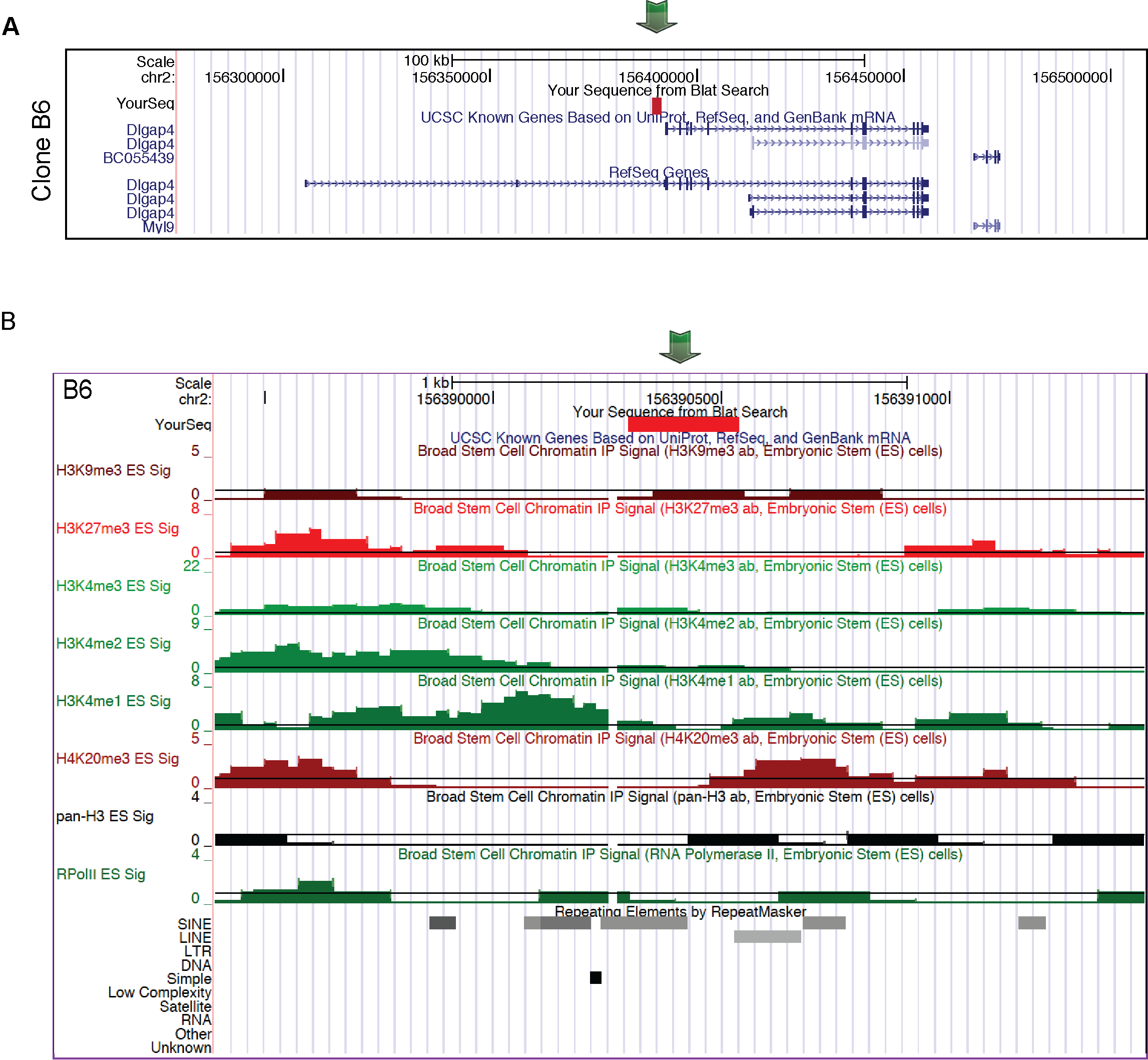

Supplement: Figure S1 — Integration site analysis of Clone B6. Integration site of Clone B6 and neighboring genes (A) with histone modifications present in ES cells and nearby repeat elements (B). (TIF) [file pone.0037130.s001.tif]

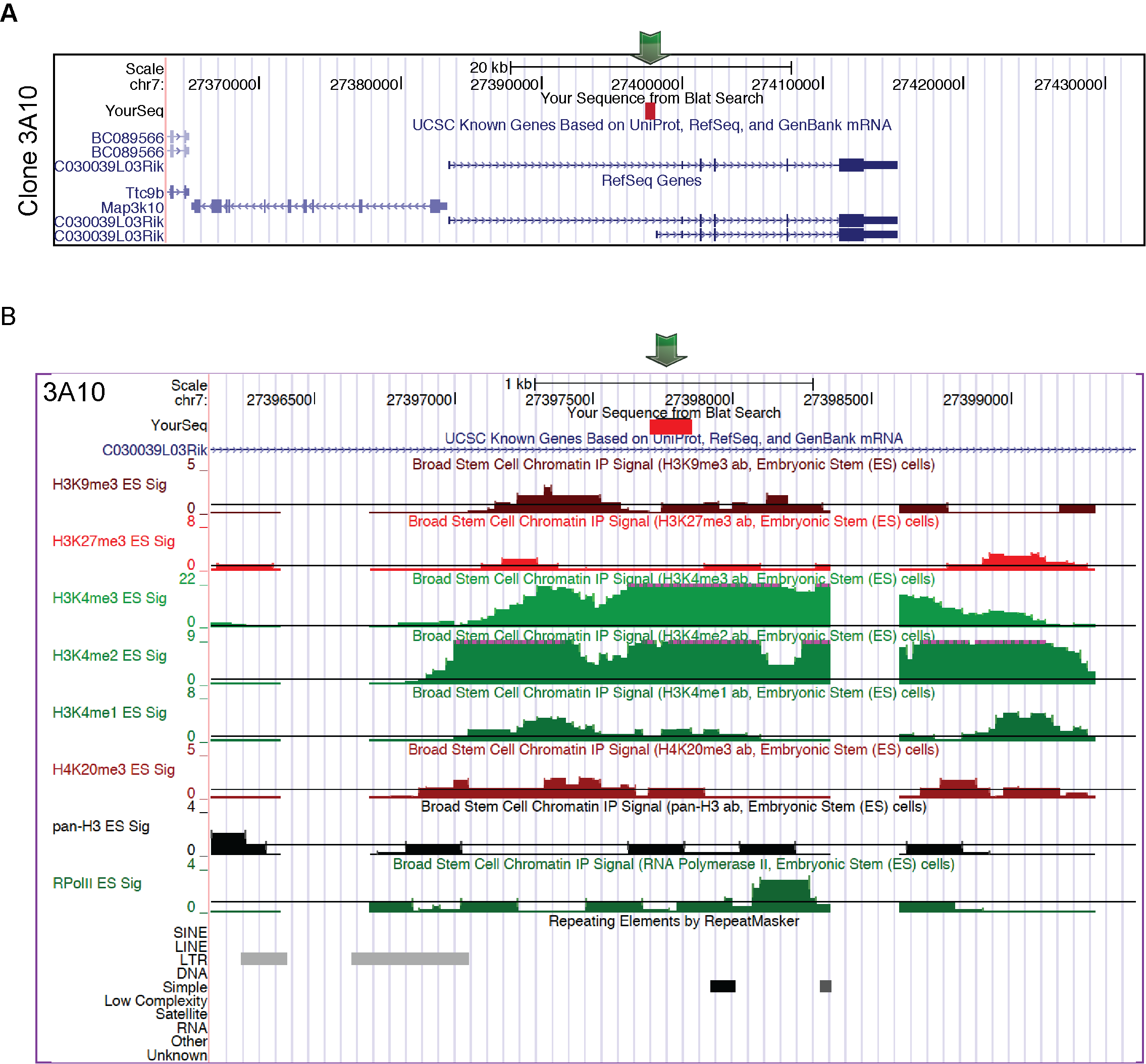

Supplement: Figure S2 — Integration site analysis of Clone 3A10. Integration site of Clone 3A10 and neighboring genes (A) with histone modifications present in ES cells and nearby repeat elements (B). (TIF) [file pone.0037130.s002.tif]

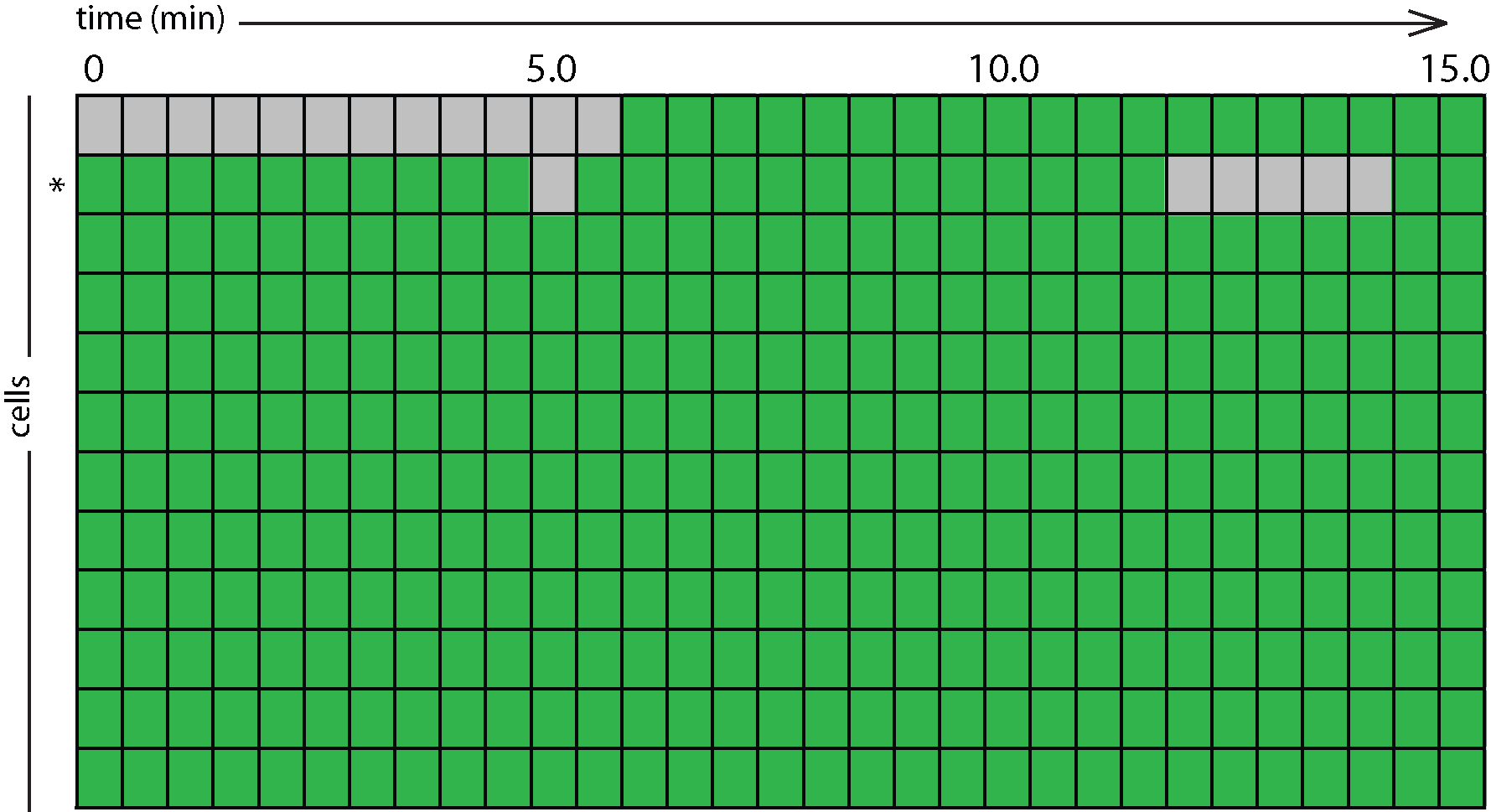

Supplement: Figure S3 — Transcriptional dynamics of Clone 3A10 imaged at 30 sec intervals. Summary of transcriptional dynamics displayed by all cells in Clone 3A10 imaged at 30 seconds intervals. Green squares indicate timepoints with detectable transcription foci and gray squares represent timepoints without transcription foci. Cell displayed in Figure 4E is marked by an asterisk. (TIF) [file pone.0037130.s003.tif]

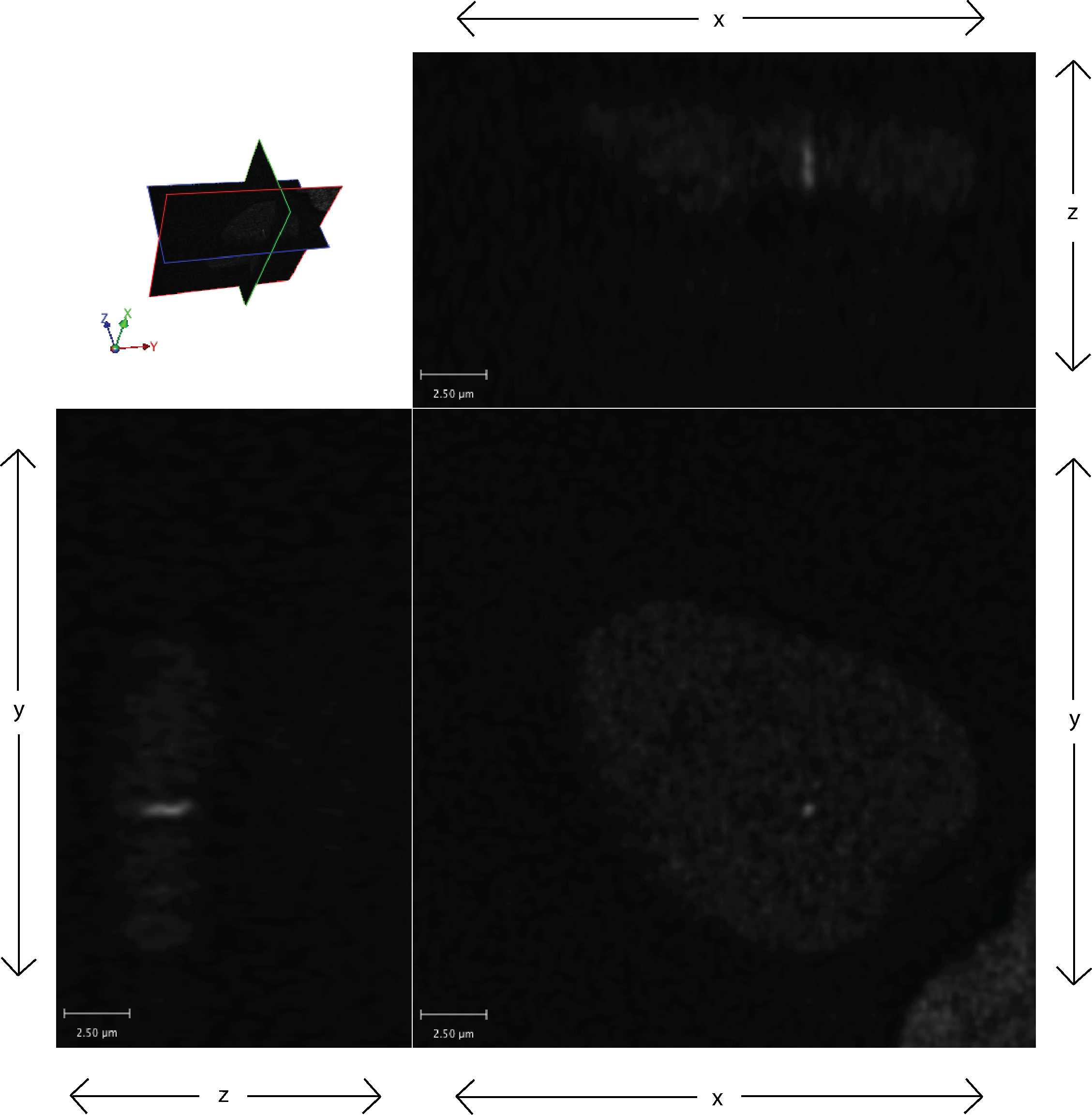

Supplement: Figure S4 — Detection of focal doublets in the z-axis. Focal doublets were detected in the z-axis, while appearing as a single focal dot in the xy-plane. (TIF) [file pone.0037130.s004.tif]
